# Does miR-618 rs2682818 variant affect cancer susceptibility? Evidence from 10 case–control studies

**DOI:** 10.1042/BSR20190741

**Published:** 2019-08-23

**Authors:** Xingliang Feng, Dan Ji, Chaozhao Liang, Song Fan

**Affiliations:** 1 Department of Urology, the First Affiliated Hospital of Anhui Medical University, Institute of Urology, Anhui Medical University, Anhui Province Key Laboratory of Genitourinary Diseases, Hefei, Anhui, China; 2 Department of Foundation Courses, Anhui Medical College, Hefei, Anhui, China

**Keywords:** cancer, meta-analysis, miR-618, polymorphism, rs2682818

## Abstract

Piles of evidence have supported the relationship between miR-618 rs2682818 polymorphism and tumorigenesis, but the conclusion remains inconsistent. In the present study, we conducted a meta-analysis to sniff out the potential risk between miR-618 rs2682818 and overall cancers. Crude odds ratios (ORs) and 95% confidence intervals (CIs) analyzed by *Z*-test were employed to estimate the potential interrelation in five genetic models. We also prospected how the rs2682818 affects the second structure of miR-618. Finally, 10 independent studies meet the enrolled criteria, along with 4099 cancer cases and 5057 healthy controls. Overall, no exceeding interrelation was sniffed out in the pooled data among five inherited models, as well as stratified analyses. Whereas, the enhanced cancer risk of miR-618 rs2682818 variant stratified by breast cancer was revealed, in heterozygote genetic model (AC vs. CC: OR = 1.291, 95%CI = 1.012–1.648, *P* = 0.040) and dominant contrast model (AA + AC vs. CC: OR = 1.280, 95%CI = 1.009–1.623, *P* = 0.042). The second structure prediction result shown that the mutant A allele might change the first stem-loop of miR-618, and the free energy of it would turn from –39.1 to –35.1 kcal/mol. All in all, our meta-analysis had successfully chased down that miR-618 rs2682818 polymorphism is not linked with overall cancer risk, but in the dominant genotype of breast cancer.

## Introduction

MicroRNAs (miRNAs), a series of 17–25 length small nucleotides, have been widely studied about its function in several types of cancer. In recent days, the method of miRNA expression profiling has been applied to reveal whether it promotes or suppresses tumorigenesis, and also exposed to be the biomarkers of predicting the occurrence or prognosis of tumors [1–5]. Although the size and number of miRNAs are less than messenger RNAs (mRNAs), they play a pivotal role in the regulation of mRNAs through binding to the 3′-UTRs of mRNA, furthermore affect the expression and function of tumor associate genes [6–8]. miRNAs could also regulate several mechanisms through the antagonization of the translation promoter of mRNA, result in the changing process of proliferation, differentiation, and apoptosis [9,10].

Recent studies have demonstrated the association between aberrant miR-618 expression and tumorigenesis; miR-618 is up-regulated in hepatocellular carcinoma, breast cancer, bladder cancer and Barrett’s esophageal cancer [11–14], while down-regulated in thyroid cancer [15]. Single nucleotide polymorphisms (SNPs) are the usual form of gene variants in genetic materials, it could also occur in the DNA sequence which encodes pre-miRNAs and miRNAs. rs2682818 polymorphism located on the precursor’s stem-loop of the miR-618 sequence, which could both affect the secondary structure of pre-miR-618 and the releasing of the mature miR-618 [16]. Whether rs2682818 polymorphism of miR-618 is associated with cancer susceptibility, we planned to verify and obtain the precise result in the current study.

## Materials and methods

We established the evaluate process of the meta-analysis along with PRISMA statement, the preferred reporting items for systematic reviews and meta-analyses compliant statement [17]. No ethics problems need to be concerned in this study, while all the data were extracted and recorded from published articles.

### Search strategy

**Figure 1:**
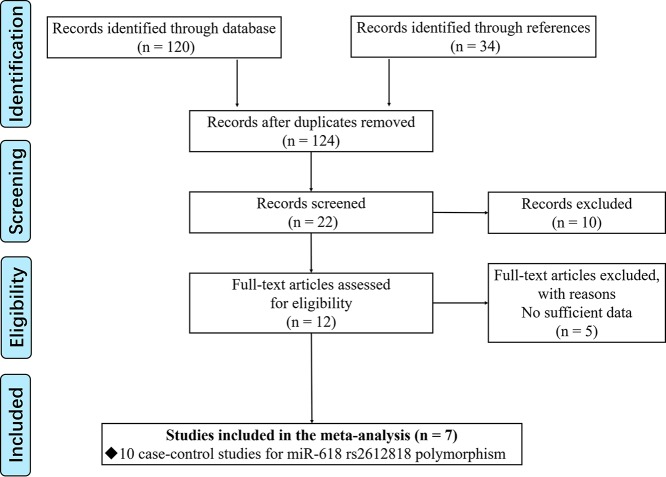
Flow chart showing the study selection process

We performed an extensive literature research about the relationship between miRNA-618 rs2682818 and cancer susceptibility up to May 2019. PubMed, Medline, Google Scholar, Wanfang and CNKI databases were all enrolled. The search items listed as following: (‘miR-618’ OR ‘microRNA-618’ OR ‘miRNA-618’ OR ‘rs2682818’) AND (‘tumor’ OR ‘cancer’ OR ‘neoplasm’) AND (‘polymorphism’ OR ‘mutation’ OR ‘variant’). The screen process of eligible studies is displayed in Figure 1.

Eligible criteria include: 1. Original studies, about cases and their matched controls, focused on the correlation of miR-618 polymorphism and cancer susceptibility. 2. Studies provided the sufficient data of SNP allele frequency in both cancer group and control group. Exclusion criteria include: 1. Studies unrelated to miRNA-618 or cancer; 2. Duplicated or repeated publication; 3. Case-only, conference abstract, review, animal or cell line articles; 4. Missing of allele frequency data.

### Extraction of data sources

Extractions of the allele frequency data from enrolled studies were completed by two authors independently. First author, published year, ethnicity of involvers, genotyping method, cancer type, and allele distribution in cancer patients and control group were all recorded. As the two reviewers independently extracted the date of different items, any inconsistent record was discussed and re-confirmed by search the original article with the two reviewers together.

### Methods of meta-analysis

We carried out the meta-analysis in pooled overall date, as well as in different stratified sub-groups. Crude odds ratios (ORs) and their 95% confidence intervals (CIs) calculated by *Z*-test were employed to estimate the potential interrelation between miR-618 rs2682818 variant and cancer risks in five common hereditary models, including allele genetic model (A vs. C), homozygote genetic model (AA vs. CC), heterozygote genetic model (AC vs. CC), recessive contrast model (AA vs. AC + CC) and dominant contrast model (AA + AC vs. CC). The model used for *Z*-test was depended upon the result of heterogeneity associated *Q*-test, if the *P* value of *Q*-test <0.1 indicated that there was meaningful heterogeneity among enrolled data, thus the random-effects model (Der Simonian and Laird method) was applied. Otherwise, the *P* value of *Q*-test ≥0.1 indicated the fixed-effects model (the Mantel–Haenszel method) [18]. Moreover, Hardy–Weinberg equilibrium (HWE) formula assessed by χ^2^ were made in control group of each study, *P*>0.05 were considered to have reliable and representative controls [19]. In order to evaluate the quality of each enrolled study, we applied Newcastle–Ottawa scale (NOS) [20].

### Evaluation of the results

Begg’s funnel plot and Egger’s test were used to appraising any publication bias in the results [21,22]. On the other way, the underlying effects of each single study to overall results were evaluated by sensitivity analyses, with the method of deletion one independent study each time. All the statistic results shown in the current study were two-tailed, while *P*-values ≤0.05 were considered to be a remarkable difference.

### *In silico* analysis of miR-618 rs2682818

In order to explore the underlying effect of rs2682818 to miR-618, we use several online tools. First, we observed the different allele frequency about rs2682818 around the world, with the help of Ensembl website based on the data extract from 1000 Genomes Project (http://asia.ensembl.org/Homo_sapiens/Variation/Population?db=core;g=ENSG00000208022;r=12:80935736-80935833;t=ENST00000385287;v=rs2682818;vdb=variation;vf=471664594#population_freq_SAS), the project unites multidisciplinary research teams from institutes around the world, including China, Italy, Japan, Kenya, Nigeria, Peru, the United Kingdom, and the United States. On the other way, the possible impact of rs2682818 located on miR-618 region on the structure of its stem-loop with the wild type and mutant allele was evaluated with an RNA structure website (http://rna.urmc.rochester.edu/RNAstructureWeb/Servers/Predict1/Predict1.html).

## Results

### Study characteristics

Along with the pre-set search items, as much as 124 articles were firstly taken into consideration from different databases. In the next two steps, we screened the relativity of each article by reading its abstract or whole manuscript. Finally, there are only seven articles met the inclusion criteria with 10 independent case–control studies [13,16,23–28]. The process of study selection is shown as a flow chart in Figure 1. Of the remaining 10 case–control studies, the details are listed in Table 1, there are concerns about 4099 cancer patients and 5057 controls. All the sources of control are population based, and accompanied by the *P* value of HWE is higher than 0.05, these two results ensured that the control group is representative and conformed to the law of genetic heredity. Meanwhile, we also assessed the quality of each study with NOS method, the result fulfilled in Supplementary Table S1 shown all the studies maintained the high quality.

**Table 1:** Characteristics of the enrolled studies on miR-618 *rs2682818* polymorphism and cancer

First author	Year	Ethnicity	Genotyping method	Source of control	Cancer type		Cases					Controls				
						Total	A allele (%)	CC	AC	AA	Total	A allele (%)	CC	AC	AA	HWE
Li et al. [25]	2011	Asian	TaqMan	PB	Hepatocellular carcinoma	339	26.0	186	130	23	352	23.7	203	131	18	0.594
Li et al. [25]	2011	Asian	TaqMan	PB	Hepatocellular carcinoma	107	26.2	55	48	4	105	27.1	57	39	9	0.533
Li et al. [25]	2011	Asian	TaqMan	PB	Nasopharyngeal carcinoma	799	26.2	444	292	63	1021	26.4	551	401	69	0.731
Wang et al. [28]	2012	Asian	TaqMan	PB	Bladder cancer	336	29.5	C = 474		A = 198	454	30.1	C = 635		A = 273	0.256
Zhang et al. [26]	2012	Asian	PCR-RFLP	PB	Breast cancer	244	25.6	132	99	13	232	24.4	130	91	11	0.325
Zhang et al. [27]	2012	Asian	PCR-RFLP	PB	Colorectal cancer	444	25.3	249	165	30	455	24.3	262	165	28	0.767
Fu et al. [16]	2014	Caucasian	MassARRAY	PB	Lymphoma	349	14.9	256	82	11	511	13.4	383	119	9	0.945
Navarro et al. [24]	2016	Caucasian	PCR	PB	Chronic lymphocytic leukemia	163	8.6	138	22	3	236	14.6	172	59	5	0.982
Morales et al. [13]	2016	Caucasian	TaqMan	PB	Breast cancer	440	9.5	359	78	3	807	7.1	699	102	6	0.290
Chen et al. [23]	2018	Asian	TaqMan	PB	Colorectal cancer	878	25.8	475	353	50	884	30.1	436	363	85	0.457

H-B: hospital based; P-B: population based; HWE: Hardy–Weinberg equilibrium, *P*>0.05 means conformed to HWE.

### Overall and subgroup analyses

**Figure 2:**
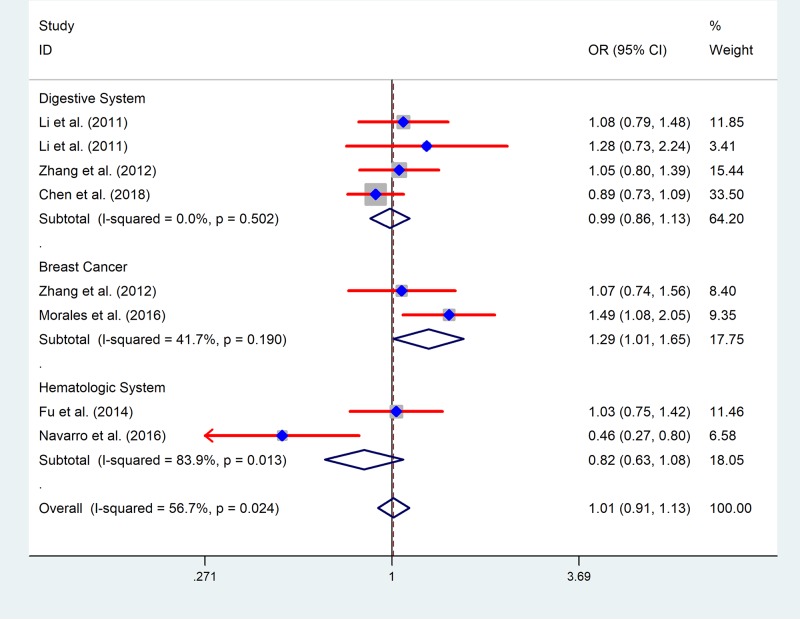
The forest plot of the meta-analysis for miR-618 rs2682818 polymorphism (AC vs. CC)

Analysis was handled to evaluate the feasible association between miR-618 rs2682818 variant and cancer risk, the results are shown in Table 2. Overall, no exceeding interrelation was sniffed out in the pooled data in all five genetic models (A vs. C: OR = 0.997, 95%CI = 0.886–1.122, *P* = 0.956; AA vs. CC: OR = 0.975, 95%CI = 0.711–1.338, *P* = 0.877; AC vs. CC: OR = 1.004, 95%CI = 0.863–1.167, *P* = 0.964; AA vs. AC + CC: OR = 0.979, 95%CI = 0.721–1.329, *P* = 0.891; AA + AC vs. CC: OR = 1.001, 95%CI = 0.860–1.165, *P* = 0.990) (Supplementary Figure S1). In the stratified calculate carried out by ethnicity, there are no vital effects of miR-618 rs2682818 variant to cancer risk on both Asian people and Caucasian people. We also tried to chase down the connection between miR-618 rs2682818 variant and different cancer type subgroup, there is no increased or decreased risk affected by it in digestive system cancer and hematologic system cancer. However, in the stratified analysis of breast cancer, we discovered an enhanced cancer risk caused by miR-618 rs2682818 variant in heterozygote genetic model (AC vs. CC: OR = 1.291, 95%CI = 1.012–1.648, *P* = 0.040) and dominant contrast model (AA + AC vs. CC: OR = 1.280, 95%CI = 1.009–1.623, *P* = 0.042) (Figure 2)**.**

**Table 2:** Results of pooled analysis for miR-618 *rs2682818* polymorphism and cancer susceptibility

Genetic model	Analysis group	*N*	*P*_H_	*P*_Z_	Effects model	OR (95% CI)
A vs. C	Overall	10	0.011	0.956	Random	0.997 (0.886–1.122)
AA vs. CC	Overall	9	0.057	0.877	Random	0.975 (0.711–1.338)
AC vs. CC	Overall	9	0.028	0.964	Random	1.004 (0.863–1.167)
AA+AC vs. CC	Overall	9	0.016	0.990	Random	1.001 (0.860–1.165)
AA vs. AC+CC	Overall	9	0.065	0.891	Random	0.979 (0.721–1.329)
A vs. C	Asian	7	0.200	0.265	Fixed	1.080 (0.899–1.297)
AA vs. CC	Asian	6	0.027	0.690	Random	0.927 (0.640–1.344)
AC vs. CC	Asian	6	0.672	0.544	Fixed	0.967 (0.868–1.077)
AA+AC vs. CC	Asian	6	0.430	0.395	Fixed	0.956 (0.863–1.060)
AA vs. AC+CC	Asian	6	0.029	0.687	Random	0.929 (0.648–1.332)
A vs. C	Caucasian	3	0.004	0.953	Random	0.987 (0.626–1.554)
AA vs. CC	Caucasian	3	0.526	0.435	Fixed	1.299 (0.674–2.504)
AC vs. CC	Caucasian	3	0.001	0.800	Random	0.929 (0.527–1.640)
AA+AC vs. CC	Caucasian	3	0.002	0.860	Random	0.953 (0.557–1.630)
AA vs. AC+CC	Caucasian	3	0.582	0.407	Fixed	0.930 (0.767–1.129)
A vs. C	Digestive system	4	0.059	0.705	Random	0.965 (0.803–1.160)
AA vs. CC	Digestive system	4	0.026	0.495	Random	0.829 (0.483–1.422)
AC vs. CC	Digestive system	4	0.502	0.847	Fixed	0.986 (0.859–1.132)
AA+AC vs. CC	Digestive system	4	0.223	0.447	Fixed	0.950 (0.833–1.084)
AA vs. AC+CC	Digestive system	4	0.037	0.442	Random	1.070 (0.528–2.168)
A vs. C	Hepatocellular carcinoma	2	0.502	0.468	Fixed	1.082 (0.875–1.338)
AA vs. CC	Hepatocellular carcinoma	2	0.119	0.789	Fixed	1.080 (0.616–1.892)
AC vs. CC	Hepatocellular carcinoma	2	0.618	0.396	Fixed	1.126 (0.856–1.481)
AA+AC vs. CC	Hepatocellular carcinoma	2	0.995	0.764	Fixed	1.121 (0.862–1.458)
AA vs. AC+CC	Hepatocellular carcinoma	2	0.090	0.764	Random	0.840 (0.270–2.618)
A vs. C	Colorectal cancer	2	0.040	0.501	Random	0.913 (0.699–1.191)
AA vs. CC	Colorectal cancer	2	0.029	0.452	Random	0.759 (0.370–1.558)
AC vs. CC	Colorectal cancer	2	0.342	0.471	Fixed	0.943 (0.804–1.106)
AA+AC vs. CC	Colorectal cancer	2	0.126	0.169	Fixed	0.899 (0.771–1.047)
AA vs. AC+CC	Colorectal cancer	2	0.043	0.428	Random	0.769 (0.401–1.473)
A vs. C	Hematologic system	2	0.009	0.554	Random	0.808 (0.398–1.639)
AA vs. CC	Hematologic system	2	0.303	0.364	Fixed	1.415 (0.669–2.994)
AC vs. CC	Hematologic system	2	0.013	0.396	Random	0.713 (0.327–1.555)
AA+AC vs. CC	Hematologic system	2	0.009	0.468	Random	0.747 (0.341–1.640)
AA vs. AC+CC	Hematologic system	2	0.393	0.312	Fixed	1.472 (0.696–3.113)
A vs. C	Breast cancer	2	0.219	0.066	Fixed	1.216 (0.987–1.497)
AA vs. CC	Breast cancer	2	0.829	0.776	Fixed	1.110 (0.542–2.270)
AC vs. CC	Breast cancer	2	0.190	0.040*	Fixed	1.291 (1.012–1.648)
AA+AC vs. CC	Breast cancer	2	0.219	0.042*	Fixed	1.280 (1.009–1.623)
AA vs. AC+CC	Breast cancer	2	0.799	0.851	Fixed	1.070 (0.528–2.168)

*P*_H_: *P* value of *Q* test for heterogeneity test; *P*_Z_*:* means statistically significant (*P* < 0.05); SCCHN: squamous cell carcinoma of the head and neck; P-B: population based; HWE: Hardy–Weinberg equilibrium; Y: polymorphisms conformed to HWE in the control group; N: polymorphisms did not conform to HWE in the control group; *P value less than 0.05 was considered as statistically significant.

### Analysis of sensitivity and publication bias

Sensitivity analysis was performed by eliminating each study one by one at a time, and the results indicated that no prominent effect from a single study would influence the stability of the above results (Supplementary Figure S2 and Table S2). As to assess the publication bias of all the enrolled studies, we deal it with statistical Egger’s test and graphical Begg’s funnel plot. We could see that the *P* value of Egger’s test in different models is higher than 0.05, and the distribution of studies in Begg’s funnel plot was symmetrical, which means there is no remarkable publication bias (Supplementary Figure S3 and Table S3).

### In silico analysis

**Figure 3:**
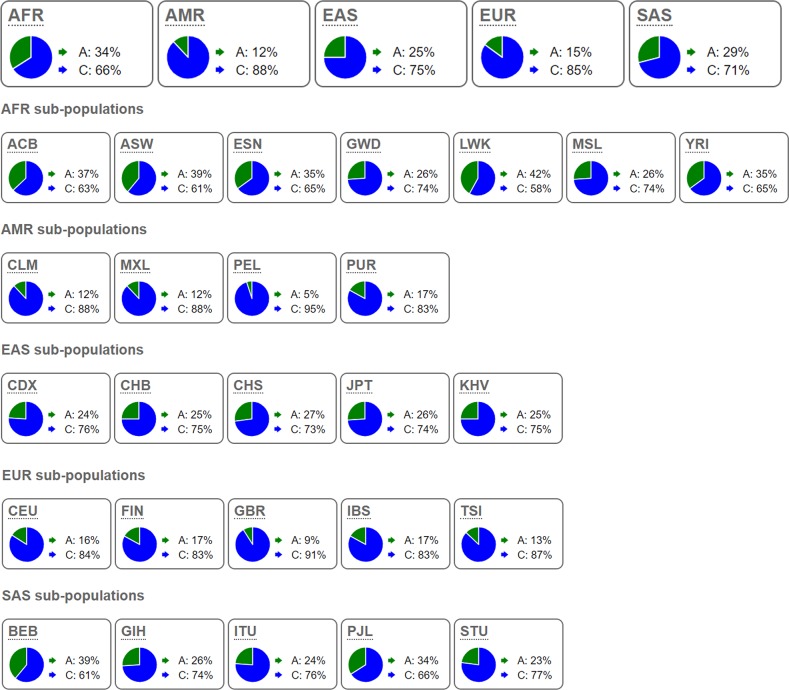
The allele frequencies of miR-618 rs2682818 in 1000 Genomes Project Phase 3 AFR: African; AMR: American; EAS: East Asian; EUR: European; SAS: South Asian; ACB: African Caribbean in Barbados; ASW: African Ancestry in Southwest US; ESN: Esan in Nigeria; GWD: Gambian in Western Division, The Gambia; LWK: Luhya in Webuye, Kenya; MSL: Mende in Sierra Leone; YRI: Yoruba in Ibadan, Nigeria; CLM: Colombian in Medellin, Colombia; MXL: Mexican Ancestry in Los Angeles, California; PEL: Peruvian in Lima, Peru; PUR: Puerto Rican in Puerto Rico; CDX: Chinese Dai in Xishuangbanna, China; CHB: Han Chinese in Beijing, China; CHS: Southern Han Chinese, China; JPT: Japanese in Tokyo, Japan; KHV: Kinh in Ho Chi Minh City, Vietnam; CEU: Utah residents with Northern and Western European ancestry; FIN: Finnish in Finland; GBR: British in England and Scotland; IBS: Iberian populations in Spain; TSI: Toscani in Italy; BEB: Bengali in Bangladesh; GIH: Gujarati Indian in Houston, TX; ITU: Indian Telugu in the UK; PJL: Punjabi in Lahore, Pakistan; STU: Sri Lankan Tamil in the UK.

**Figure 4:**
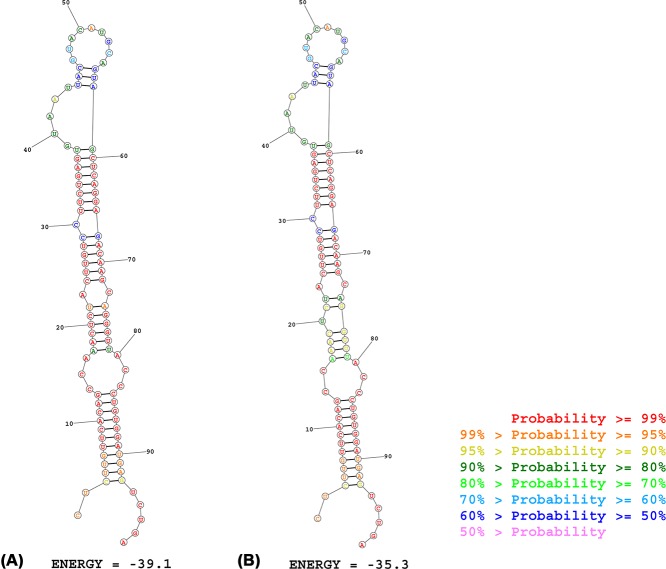
Predicted the secondary structure of miR-618 with the wild C allele (A) and mutant A allele (B)

The results obtained from online tools make us understand how the rs2682818 variant affects miR-618 more deeply. From the data extracted from 1000 Genomes Project, we learned that the distribution of C allele and A allele in rs2682818 is inconsistent in different zones. As shown in Figure 3 and Supplementary Table S4, the rate of A allele in AMR and EUR is about 5–17%, consist with the data of three Caucasian base studies in current study (Fu et al.: 13.4%; Navarro et al.: 14.6%; Morales et al.: 7.1%). On the meanwhile, the rate of A allele in EAS and SAS is about 23–39%, also consist with the data of seven Caucasian base studies in the current study (Li et al. (a): 23.7%; Li et al. (b): 27.1%; Li et al. (c): 26.4%; Wang et al.: 30.1%; Zhang et al. (a): 24.4%; Zhang et al. (b): 24.3%; Chen et al.: 30.1%) (Supplementary Table S4). The allele frequency obtained from 1000 Genomes Project supported that all the enrolled studies comforted to nature inheritance in different zones and ethnicities. The second structure of miR-618 might be changed with or without the mutant of rs2682818. As illustrated in Figure 4, the mutant A allele might change the first stem-loop of miR-618, and the free energy of it would turn from −39.1 to −35.1 kcal/mol.

## Discussion

miR-618, the 98-nucleotide composed small molecule is the single transcription product of MIR618, which located on chromosome 12. In recent days, miR-618 has been reported associated with different types of cancer. Song et al. [29] reported that miR-618 could induce the transition of prostate cell from epithelial to mesenchymal through the target combined way to forkhead box p2 (FOXP2), finally lead to the inhibition of cell migration and invasion. On the meanwhile, Ivanovic et al. [30] found out that the overexpression of miR-618 could suppress the function of MMP-9 to prevent the progress of tumorigenesis, on the other hand, they also obtained the links that the expression level of miR-618 is negatively associated with the high Gleason score and advanced stages. Shi et al. [31] also revealed that the expression level of miR-618 in gastric cancer tissues is down-regulated compared with the adjacent non-tumor tissues, while overexpression of miR-618 could suppress the migration and invasion capacity of gastric cells.

Cause miR-618 plays an essential role in suppressing mRNA function through the combined effect of its second structure, the rs2682818 polymorphism on it is part of its precursor’s stem-loop sequence. Fu et al. [16] reported that rs2682818 increased the susceptibility of follicular lymphoma (OR = 1.65, 95% CI = 1.05–2.50), and the variant A allele is associated with the decrease the level of mature miR-618. In the contrast, Navarro et al. [24] gave the evidence that rs2682818 polymorphism plays a protective role in chronic lymphocytic leumkemia (CLL) (OR = 0.49, 95% CI = 0.29–0.81). For colorectal cancer (CRC), Chen et al. [23] sniff out that individuals who carry the AA or AC+AA genotype take along a lower CRC risk than CC (AA vs. CC: OR = 0.54, 95%CI = 0.37–0.79; AC+AA vs. CC: OR = 0.82, 95% CI = 0.68–0.99). Whereas, Wang et al. [28] presented the result that rs2682818 is not associated with the sensibility of CRC (AC vs. CC: OR = 1.03, 95%CI = 0.77–1.36; OR = 1.05, AA vs. CC: 95% CI = 0.60–1.84). Due to the misleading puzzle about rs2682818 and cancer risks, we managed this meta-analysis to remove the gaps. Results from our study show that rs2682818 of miR-618 is not associated with cancer sensibility, but it may act as the promoter of breast cancer. The *in silico* analysis from 1000 Genomes Project supported that all the enrolled studies comforted to the nature inheritance genetic rate in different zones and ethnicities. The second structure of miR-618 might be changed by rs2682818 polymorphism with the mutant A allele located at the first stem-loop of miR-618.

This study is the first meta-analysis concerned about the relationship between miR-618 rs2682818 polymorphism and cancer risk, we obtain some meaningful results, but the results should also be cautiously handled. There is some limitation that should not be covered up. First of all, the insufficient capacity of data occurred in the subgroup analysis, it might cause slight effects on the results of cancer risks. Besides, we ignored the feasible effect of complex factors, which rejects us to further evaluate the influence of gene–environment relations. On the meanwhile, there are also some obviously advantages. On the one hand, this is the first meta-analysis to talk about whether miR-618 rs2682818 would influence tumorigenicity. On the other hand, we completed a comprehensive literature search to enroll the correct studies, and NOS scale was used to eliminate the low-quality studies, so the results are reliable and unmistakable. What’s more, as the results showing, miR-618 rs2682818 is associated with the tumorigenesis of breast cancer, it could be a potential biomarker to remind people who with the polymorphism of rs2682818 pay more attention to the occurrence of breast cancer, and solve the problem as soon as possible.

In conclusion, our meta-analysis successfully chased down that miR-618 rs2682818 polymorphism is not linked with overall cancer risk, but in the dominant genotype of breast cancer.

## Supporting information

**Supplementary Figure S1:** 

**Supplementary Figure S2:** 

**Supplementary Figure S3:** 

**Supplementary Table S1:** Methodological quality of the includeds studies according to the Newcastle-Ottawa Scale.

**Supplementary Table S2:** Details of the sensitivity analyses for *miR-618 rs2612818* polymorphism and cancer risk.

**Supplementary Table S3:** *P* values of the Egger’s test for *miR-618 rs2612818* polymorphism.

**Supplementary Table S4:** The allele frequencies of *miR-618 rs2612818* in 1000 Genomes Project Phase 3

## Abbreviations

CLL: chronic lymphocytic leukemia
CRC: colorectal cancer
FOXP2: forkhead box p2
HWE: Hardy–Weinberg equilibrium
miRNA: microRNA
mRNA: messenger RNA
NOS: Newcastle–Ottawa scale
ORs: odds ratios
SNP: single nucleotide polymorphism
