# Supplementary material for: Does miR-618 rs2682818 variant affect cancer susceptibility? Evidence from 10 case–control studies
Source: Biosci Rep. 2019 Aug 23;39(8):BSR20190741. doi: 10.1042/BSR20190741 (PMC6706600; doi:10.1042/BSR20190741)
Supplement: Supplementary file 1 [file bsr20190741_Supp1.pdf]

Supplementary tables and figures

Whether miR-618 rs612818 variant affect cancer susceptibility: evidence from 10 case-control studies

Xingliang Feng[1], Dan Ji[2], Chaozhao Liang[1*], Song Fan[1*]

[1]Department of Urology, the First Affiliated Hospital of Anhui Medical; University, Institute of Urology, Anhui Medical University , Anhui Province Key Laboratory of Genitourinary Diseases, Anhui Medical University, Hefei , Anhui, China.

[2]Department of foundation courses, Anhui Medical University

Correspondence:

Prof. Song Fan

Department of Urology, The First Affiliated Hospital of Anhui Medical University, Institute of Urology, Anhui Medical University, Anhui Province Key Laboratory of Genitourinary Diseases, Anhui Medical University, No. 218, Jixi Road, Hefei, 230022, Anhui, China.

Email: songfandoctor@gmail.com

Prof. Chaozhao Liang

Department of Urology, The First Affiliated Hospital of Anhui Medical University, Institute of Urology, Anhui Medical University, Anhui Province Key Laboratory of Genitourinary Diseases, Anhui Medical University, No. 218, Jixi Road, Hefei, 230022, Anhui, China.

Email: liang_chaozhao@ahmu.edu.cn

Running Title: Association between *miR-618 rs2612818* polymorphism and cancer risk.

Supplementary table 1. Methodological quality of the included studies according to the Newcastle-Ottawa Scale.

SNP	Author	Year	Adequacy of Case Definition	Representativeness of the Cases	Selection of Controls	Definition of Controls	Comparability Cases/Controls	Ascertainment of Exposure	Same Method of Ascertainment	Non-response rate
rs2612818	Li et al.	2011	*	*	*	*	*	*	*	NA
rs2612818	Wang et al.	2012	*	*	*	*	*	*	*	NA
rs2612818	Zhang et al.	2012	*	*	*	*	**	*	*	NA
rs2612818	Fu et al.	2014	*	*	*	*	*	*	*	NA
rs2612818	Navarro et al.	2016	*		*	*		*	*	NA
rs2612818	Morales et al.	2016	*	*	*	*	**	*	*	NA
rs2612818	Chen et al.	2018	*	*	*	*	*	*	*	NA

This table identifies “high” quality choices with a “star”. A study can be awarded a maximum of 1 star for each numbered item within the Selection and Exposure categories. A maximum of 2 stars can be given for Comparability. *, Yes; NA, not applicable. (http://www.ohri.ca/programs/clinical epidemiology/oxford.htm).

Supplementary table 2. Details of the sensitivity analyses for *miR-618 rs2612818* polymorphism and cancer risk.

Comparison	Study omitted	Estimate (95% Confident Interval)	Effect Model
A vs. C	Li et al. (2011)	0.98244447(0.86461496-1.1163317)	Random
	Li et al. (2011)	0.9995321(0.88136935-1.1335367)	
	Li et al. (2011)	0.99812555(0.86618596-1.1501625)	
	Wang et al. (2012)	1.0001068(0.87455195-1.1436869)	
	Zhang et al. (2012)	0.98997068(0.8705346-1.1257932)	
	Zhang et al. (2012)	0.98902851(0.86622745-1.1292385)	
	Fu et al. (2014)	0.98374742(0.86644101-1.1169358)	
	Navarro et al. (2016)	1.0207899(0.91829252-1.1347276)	
	Morales et al. (2016)	0.96599936(0.86515617-1.0785968)	
	Chen et al. (2018)	1.0367316(0.92938352-1.156479)	
	Combined	0.99665673(0.8855405-1.1217156)	
AA vs. CC	Li et al. (2011)	0.92607081(0.65752488-1.304296)	Random
	Li et al. (2011)	1.0173988(0.73469293-1.4088886)	
	Li et al. (2011)	0.94666171(0.64687091-1.3853899)	
	Zhang et al. (2012)	0.95998102(0.67688984-1.3614675)	
	Zhang et al. (2012)	0.95498514(0.66061336-1.38053)	
	Fu et al. (2014)	0.91860098(0.66742694-1.2642998)	
	Navarro et al. (2016)	0.98951745(0.70665175-1.3856115)	
	Morales et al. (2016)	0.97803891(0.69729382-1.3718178)	
	Chen et al. (2018)	1.1489894(0.90558553-1.4578156)	
	Combined	0.97536646(0.71080236-1.3384026)	
AC vs. CC	Li et al. (2011)	0.993662(0.83861649-1.1773727)	Random
	Li et al. (2011)	0.98945022(0.84481949-1.1588414)	
	Li et al. (2011)	1.0229887(0.85552865-1.2232273)	
	Zhang et al. (2012)	0.99655646(0.84332883-1.1776247)	
	Zhang et al. (2012)	0.99661452(0.83802658-1.1852136)	
	Fu et al. (2014)	1.0001394(0.84336382-1.1860583)	
	Navarro et al. (2016)	1.0320474(0.9171344-1.1613585)	
	Morales et al. (2016)	0.95463401(0.83816516-1.0872869)	

	Chen et al. (2018)	1.025502(0.85911518-1.2241131)	
	Combined	1.0035064(0.86281909-1.1671336)	
AA+AC vs. CC	Li et al. (2011)	0.98631877(0.83355123-1.1670846)	Random
	Li et al. (2011)	0.99378771(0.84566242-1.1678585)	
	Li et al. (2011)	1.0122422(0.84219897-1.2166178)	
	Zhang et al. (2012)	0.99265063(0.83995807-1.1731006)	
	Zhang et al. (2012)	0.9921385(0.83423871-1.1799246)	
	Fu et al. (2014)	0.99054599(0.83626443-1.1732908)	
	Navarro et al. (2016)	1.0353016(0.91258597-1.1745188)	
	Morales et al. (2016)	0.95535421(0.83360338-1.0948873)	
	Chen et al. (2018)	1.0386996(0.88288075-1.2220188)	
	Combined	1.0010053(0.86014524-1.1649331)	
AA vs. AC+CC	Li et al. (2011)	0.93451869(0.66838723-1.3066155)	Random
	Li et al. (2011)	1.0236579(0.75279146-1.3919865)	
	Li et al. (2011)	0.93694293(0.6543535-1.3415716)	
	Zhang et al. (2012)	0.9663009(0.68829137-1.356602)	
	Zhang et al. (2012)	0.96140999(0.67139369-1.3767022)	
	Fu et al. (2014)	0.92516363(0.67897832-1.2606113)	
	Navarro et al. (2016)	0.98595256(0.71136266-1.3665358)	
	Morales et al. (2016)	0.98380953(0.70924634-1.3646615)	
	Chen et al. (2018)	1.1542377(0.91395056-1.4576989)	
	Combined	0.97879839(0.72076184-1.3292134)	

Supplementary table 3. *P* values of the Egger's test for *miR-618 rs2612818* polymorphism.

Polymorphisms	Subgroup	Egger's test P > |t|
rs2612818	Overall	0.617
	Asian	0.251
	Caucasian	0.279
	Digestive System	0.403

Supplementary table 4. The allele frequencies of *miR-618 rs2612818* in 1000 Genomes Project Phase 3

Population	Allele: frequency (count)	Genotype: frequency (count)
ALL;1000GENOMES:phase_3:ALL	A: 0.242 (1214) C: 0.758 (3794)	A|A: 0.066 (165) A|C: 0.353 (884) C|C: 0.581 (1455)
AFR;1000GENOMES:phase_3:AFR	A: 0.340 (449) C: 0.660 (873)	A|A: 0.120 (79) A|C: 0.440 (291) C|C: 0.440 (291)
ACB;1000GENOMES:phase_3:ACB	A: 0.370 (71) C: 0.630 (121)	A|A: 0.104 (10) A|C: 0.531 (51) C|C: 0.365 (35)
ASW;1000GENOMES:phase_3:ASW	A: 0.393 (48) C: 0.607 (74)	A|A: 0.197 (12) A|C: 0.393 (24) C|C: 0.410 (25)
ESN;1000GENOMES:phase_3:ESN	A: 0.348 (69) C: 0.652 (129)	A|A: 0.121 (12) A|C: 0.455 (45) C|C: 0.424 (42)
GWD;1000GENOMES:phase_3:GWD	A: 0.257 (58) C: 0.743 (168)	A|A: 0.071 (8) A|C: 0.372 (42) C|C: 0.558 (63)
LWK;1000GENOMES:phase_3:LWK	A: 0.419 (83) C: 0.581 (115)	A|A: 0.182 (18) A|C: 0.475 (47) C|C: 0.343 (34)
MSL;1000GENOMES:phase_3:MSL	A: 0.259 (44) C: 0.741 (126)	A|A: 0.094 (8) A|C: 0.329 (28) C|C: 0.576 (49)
YRI;1000GENOMES:phase_3:YRI	A: 0.352 (76) C: 0.648 (140)	A|A: 0.102 (11) A|C: 0.500 (54) C|C: 0.398 (43)
AMR;1000GENOMES:phase_3:AMR	A: 0.118 (82) C: 0.882 (612)	A|A: 0.012 (4) A|C: 0.213 (74) C|C: 0.775 (269)
CLM;1000GENOMES:phase_3:CLM	A: 0.117 (22) C: 0.883 (166)	A|A: 0.011 (1) A|C: 0.213 (20) C|C: 0.777 (73)
MXL;1000GENOMES:phase_3:MXL	A: 0.117 (15) C: 0.883 (113)	A|C: 0.234 (15) C|C: 0.766 (49)
PEL;1000GENOMES:phase_3:PEL	A: 0.053 (9) C: 0.947 (161)	A|C: 0.106 (9) C|C: 0.894 (76)
PUR;1000GENOMES:phase_3:PUR	A: 0.173 (36) C: 0.827 (172)	A|A: 0.029 (3) A|C: 0.288 (30) C|C: 0.683 (71)
EAS;1000GENOMES:phase_3:EAS	A: 0.253 (255) C: 0.747 (753)	A|A: 0.071 (36) A|C: 0.363 (183) C|C: 0.565 (285)
CDX;1000GENOMES:phase_3:CDX	A: 0.237 (44) C: 0.763 (142)	A|A: 0.065 (6) A|C: 0.344 (32) C|C: 0.591 (55)
CHB;1000GENOMES:phase_3:CHB	A: 0.248 (51) C: 0.752 (155)	A|A: 0.068 (7) A|C: 0.359 (37) C|C: 0.573 (59)
CHS;1000GENOMES:phase_3:CHS	A: 0.271 (57) C: 0.729 (153)	A|A: 0.095 (10) A|C: 0.352 (37) C|C: 0.552 (58)
JPT;1000GENOMES:phase_3:JPT	A: 0.260 (54) C: 0.740 (154)	A|A: 0.067 (7) A|C: 0.385 (40) C|C: 0.548 (57)
KHV;1000GENOMES:phase_3:KHV	A: 0.247 (49) C: 0.753 (149)	A|A: 0.061 (6) A|C: 0.374 (37) C|C: 0.566 (56)
EUR;1000GENOMES:phase_3:EUR	A: 0.145 (146) C: 0.855 (860)	A|A: 0.020 (10) A|C: 0.250 (126) C|C: 0.730 (367)
CEU;1000GENOMES:phase_3:CEU	A: 0.162 (32) C: 0.838 (166)	A|A: 0.030 (3) A|C: 0.263 (26) C|C: 0.707 (70)
FIN;1000GENOMES:phase_3:FIN	A: 0.172 (34) C: 0.828 (164)	A|A: 0.030 (3) A|C: 0.283 (28) C|C: 0.687 (68)
GBR;1000GENOMES:phase_3:GBR	A: 0.093 (17) C: 0.907 (165)	A|A: 0.011 (1) A|C: 0.165 (15) C|C: 0.824 (75)
IBS;1000GENOMES:phase_3:IBS	A: 0.168 (36) C: 0.832 (178)	A|C: 0.336 (36) C|C: 0.664 (71)
TSI;1000GENOMES:phase_3:TSI	A: 0.126 (27) C: 0.874 (187)	A|A: 0.028 (3) A|C: 0.196 (21) C|C: 0.776 (83)
SAS;1000GENOMES:phase_3:SAS	A: 0.288 (282) C: 0.712 (696)	A|A: 0.074 (36) A|C: 0.429 (210) C|C: 0.497 (243)

BEB;1000GENOMES:phase_3:BEB	A: 0.390 (67) C: 0.610 (105)	A|A: 0.128 (11) A|C: 0.523 (45) C|C: 0.349 (30)
GIH;1000GENOMES:phase_3:GIH	A: 0.262 (54) C: 0.738 (152)	A|A: 0.039 (4) A|C: 0.447 (46) C|C: 0.515 (53)
ITU;1000GENOMES:phase_3:ITU	A: 0.235 (48) C: 0.765 (156)	A|A: 0.059 (6) A|C: 0.353 (36) C|C: 0.588 (60)
PJL;1000GENOMES:phase_3:PJL	A: 0.344 (66) C: 0.656 (126)	A|A: 0.104 (10) A|C: 0.479 (46) C|C: 0.417 (40)
STU;1000GENOMES:phase_3:STU	A: 0.230 (47) C: 0.770 (157)	A|A: 0.049 (5) A|C: 0.363 (37) C|C: 0.588 (60)

AFR: African; AMR: American; EAS: East Asian; EUR: European; SAS: South Asian; ACB: African Caribbean in Barbados; ASW: African Ancestry in Southwest US; ESN: Esan in Nigeria; GWD: Gambian in Western Division, The Gambia; LWK: Luhya in Webuye, Kenya; MSL: Mende in Sierra Leone; YRI: Yoruba in Ibadan, Nigeria; CLM: Colombian in Medellin, Colombia; MXL: Mexican Ancestry in Los Angeles, California; PEL: Peruvian in Lima, Peru; PUR: Puerto Rican in Puerto Rico; CDX: Chinese Dai in Xishuangbanna, China; CHB: Han Chinese in Bejing, China; CHS: Southern Han Chinese, China; JPT: Japanese in Tokyo, Japan; KHV: Kinh in Ho Chi Minh City, Vietnam; CEU: Utah residents with Northern and Western European ancestry; FIN: Finnish in Finland; GBR: British in England and Scotland; IBS: Iberian populations in Spain; TSI: Toscani in Italy; BEB: Bengali in Bangladesh; GIH: Gujarati Indian in Houston, TX; ITU: Indian Telugu in the UK; PJL: Punjabi in Lahore, Pakistan; STU: Sri Lankan Tamil in the UK;

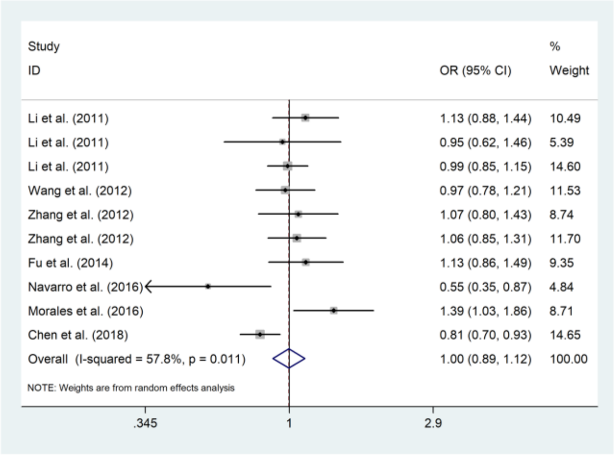

C vs. A

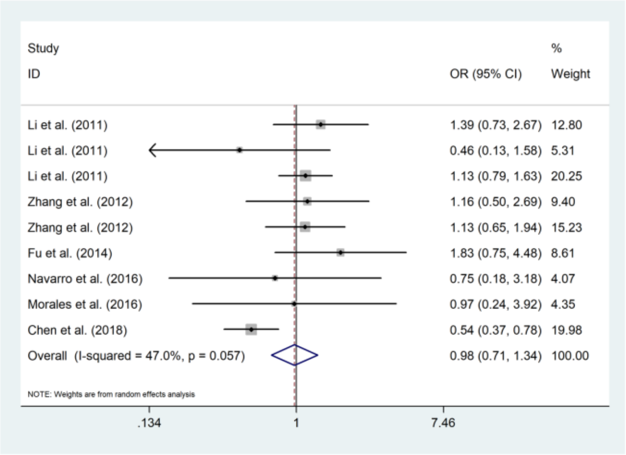

CC vs. AA

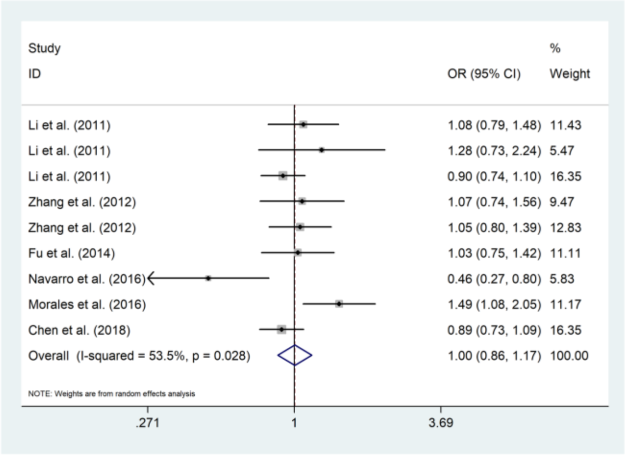

CA vs. AA

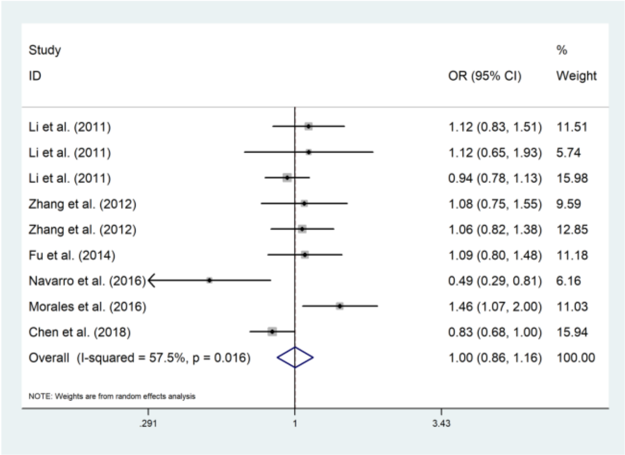

CC+CA vs. AA

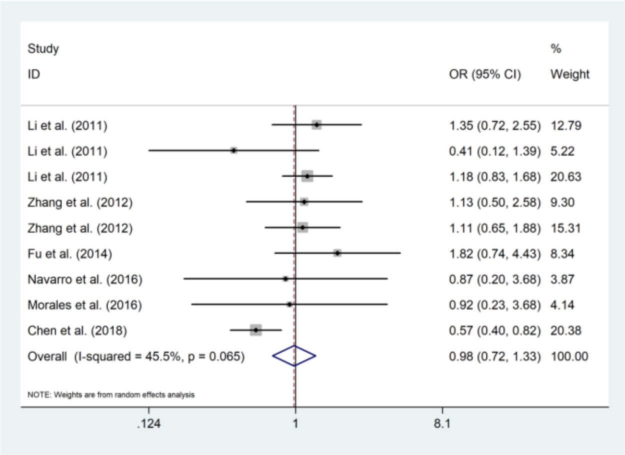

CC vs. CA+AA

Fig.S1

Meta-analysis of the association between *miR-618 rs2612818* polymorphism and overall cancer risk.

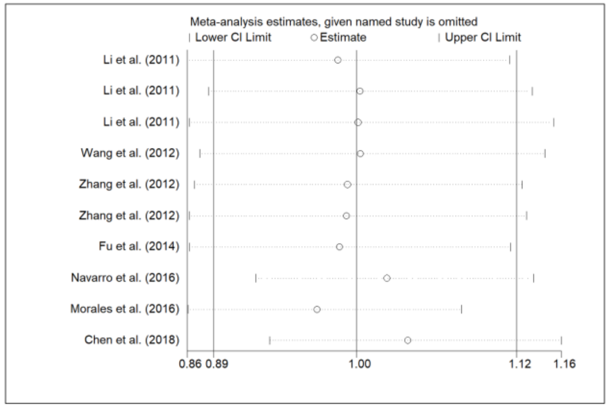

C vs. A

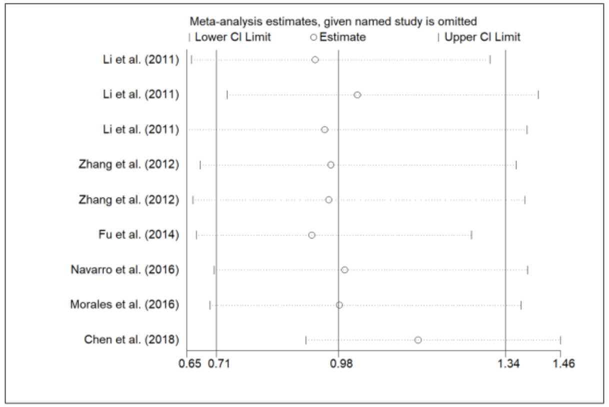

CC vs. AA

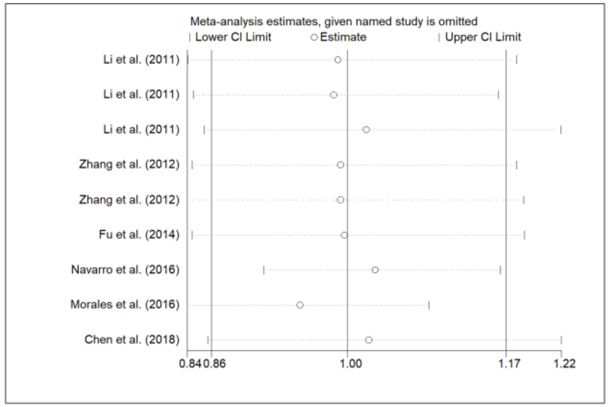

CA vs. AA

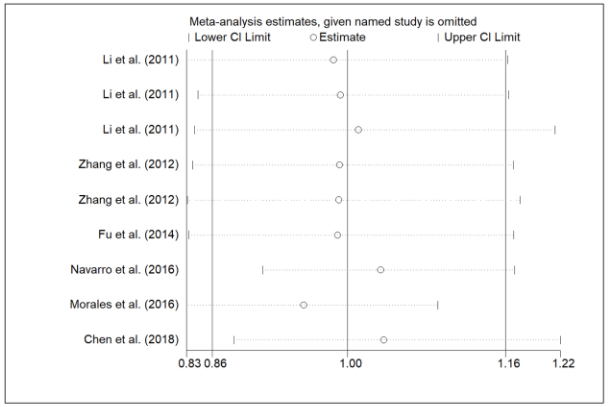

CC+CA vs. AA

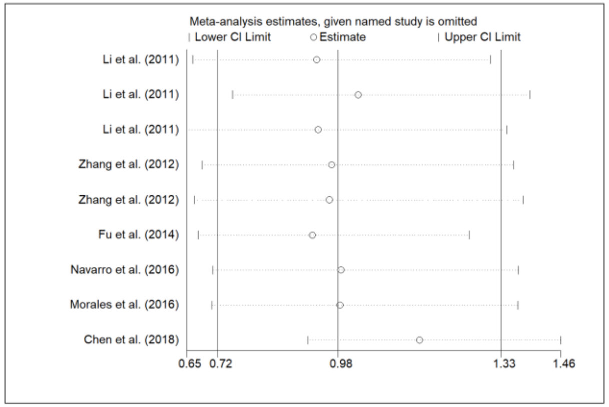

CC vs. CA+AA

Fig.S2

Sensitivity analysis of *miR-618 rs2612818* polymorphism and overall cancer susceptibility

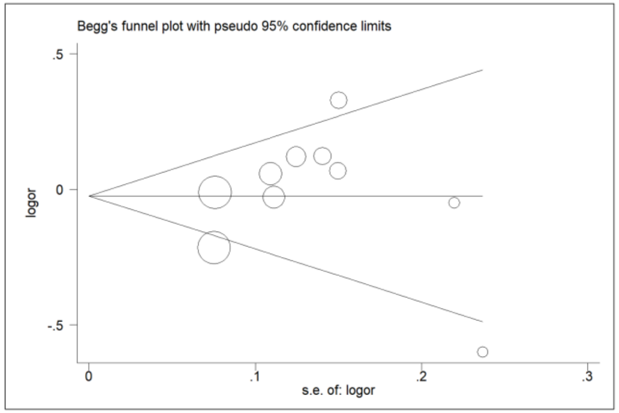

C vs. A

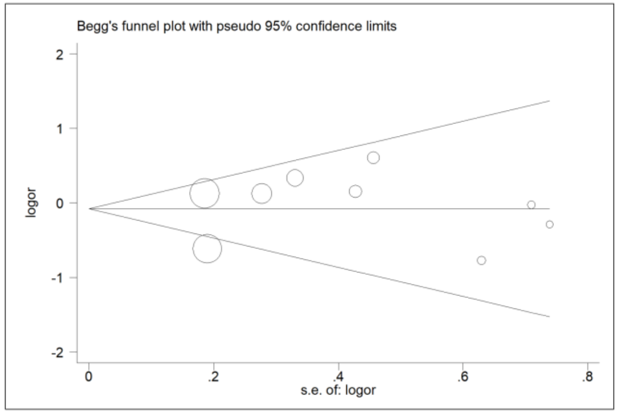

CC vs. AA

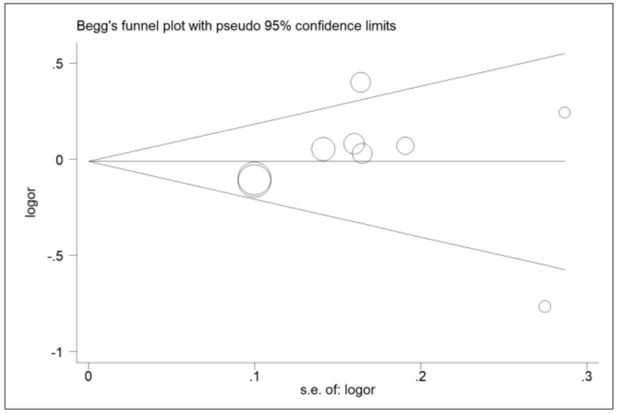

CA vs. AA

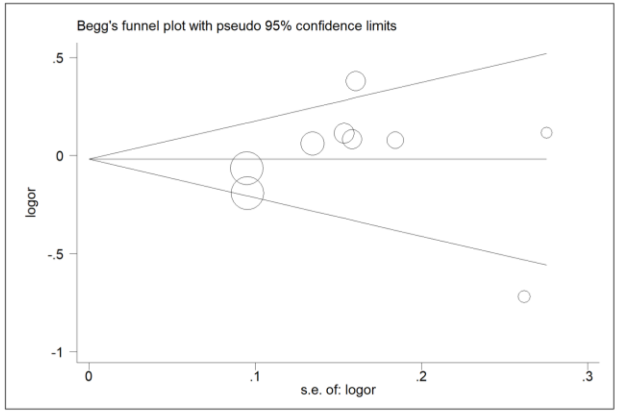

CC+CA vs. AA

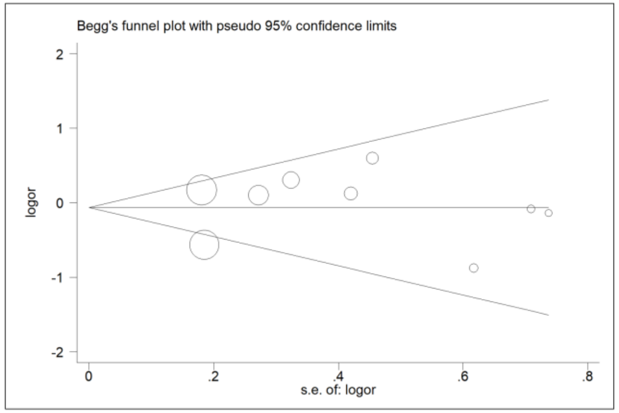

CC vs. CA+AA

Fig.S3

Begg's funnel plot for publication bias test under *miR-618 rs2612818*. The x-axis is log (OR), and the y-axis is natural logarithm of OR. The horizontal line in the figure represents the overall estimated log (OR). The two diagonal lines indicate the pseudo 95% confidence limits of the effect estimate.
